# *N*-(Hydroxymethyl)ibogaine

**DOI:** 10.1107/S1600536808025324

**Published:** 2008-08-09

**Authors:** Raoudha Mezghani Jarraya, Amira Bouaziz, Besma Hamdi, Abdelhamid Ben Salah, Mohamed Damak

**Affiliations:** a Laboratoire de Chimie des Substances Naturelles, Faculté des Sciences de Sfax, BP 1171, 3000 Sfax, Tunisia; b Laboratoire des Sciences de Materiaux et d’Environnement, Faculté des Sciences de Sfax, BP 1171, 3000 Sfax, Tunisia

## Abstract

The title compound (systematic name: 16-hydroxy­methyl-12-methoxy­ibogamine), C_21_H_28_N_2_O_2_, was prepared by reaction of ibogaine with a formaldehyde–acetic acid solution (pH = 4). The crystal structure of this new product, belonging to the iboga indole family, is stabilized by an inter­molecular O—H⋯N hydrogen bond. The identity of the compound was confirmed by one- and two-dimensional NMR spectroscopic techniques.

## Related literature

For related literature on ibogaine and its derivatives, see: Alper *et al.* (2008 ▶); Levant & Pazdernik (2004 ▶); Maisonneuve *et al.* (1991 ▶); Soriano-García (1992 ▶).
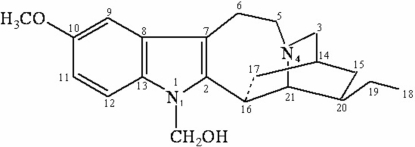


## Experimental

### 

#### Crystal data

C_21_H_28_N_2_O_2_
*M* *_r_* = 340.45
Orthorhombic, 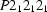

*a* = 8.4990 (10) Å
*b* = 10.2537 (11) Å
*c* = 20.676 (3) Å
*V* = 1801.8 (4) Å^3^
*Z* = 4
Mo *K*α radiation
μ = 0.08 mm^−1^
*T* = 293 (2) K
0.47 × 0.33 × 0.26 mm

#### Data collection

Bruker Kappa APEXII CCD diffractometer
Absorption correction: multi-scan (Coppens *et al.*, 1965 ▶) *T*_min_ = 0.962, *T*_max_ = 0.981
9906 measured reflections
2131 independent reflections
1225 reflections with *I* > 2σ(*I*)
*R*_int_ = 0.079

#### Refinement

*R*[*F* ^2^ > 2σ(*F* ^2^)] = 0.049
*wR*(*F* ^2^) = 0.123
*S* = 1.00
2131 reflections
228 parameters
H-atom parameters constrained
Δρ_max_ = 0.25 e Å^−3^
Δρ_min_ = −0.17 e Å^−3^

### 

Data collection: *APEX2* (Bruker, 2004 ▶); cell refinement: *SAINT* (Bruker, 1998 ▶); data reduction: *SAINT*; program(s) used to solve structure: *SHELXS97* (Sheldrick, 2008 ▶); program(s) used to refine structure: *SHELXL97* (Sheldrick, 2008 ▶); molecular graphics: *ORTEP-3* (Farrugia, 1997 ▶); software used to prepare material for publication: *WinGX* (Farrugia, 1999 ▶).

## Supplementary Material

Crystal structure: contains datablocks I, global. DOI: 10.1107/S1600536808025324/zl2132sup1.cif

Structure factors: contains datablocks I. DOI: 10.1107/S1600536808025324/zl2132Isup2.hkl

Additional supplementary materials:  crystallographic information; 3D view; checkCIF report

## Figures and Tables

**Table 1:** Hydrogen-bond geometry (Å, °)

*D*—H⋯*A*	*D*—H	H⋯*A*	*D*⋯*A*	*D*—H⋯*A*
O2—H2⋯N4^i^	0.82	2.10	2.825 (3)	148

Symmetry code: (i) 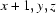.

**Fig. 1.**
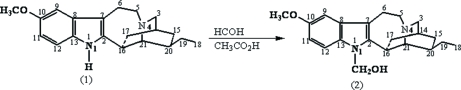
Chemical pathway of the formation of the N-hydroxymethylene ibogaine (2).

**Fig. 2.**
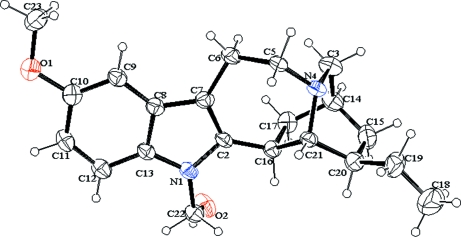
ORTEP drawing of the title compound with the atom-labelling scheme. Ellispsoids are drawn at the 30% probability level. H atoms are represented as small spheres of arbitrary radii.

**Fig. 3.**
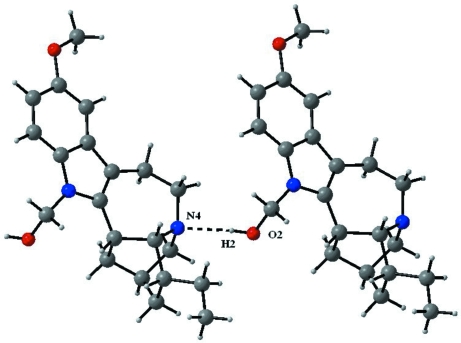
Partial packing view showing the formation of intermolecular O–H···N hydrogen bonds.

**Fig. 4.**
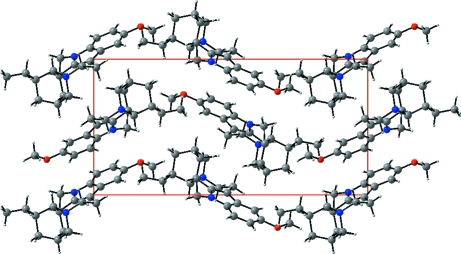
The crystal paking of the N-hydroxymethylene ibogaine structure along [001].

## supplementary crystallographic information

### Comment

Ibogaine is the main alkaloid found in the root bark of Tabernanthe iboga (Apocynaceae family), a shrub native to equatorial Africa. Its crystal structure was previously established by *M*. Soriano-García (1992). It is used, at low doses, to produce increased energy, arousal, and appetite and at high doses, for its hallucinogenic properties (Maisonneuve *et al.*, 1991) and it has been claimed to be effective in abolishing drug craving in heroin and cocaine addicts (Levant & Pazdernik, 2004).

Ibogaine is a psychostimulant of interest as an ethnopharmacological prototype for experimental investigation and possible rational pharmaceutical development (Alper *et al.*, 2008). In this context and in order to prepare other substitutes, we realised the reaction of ibogaine with a formaldehyde-acetic acid solution (pH= 4). This reaction led to 47% of the title compound (Fig. 1).

The current study describes the preparation and the structure elucidation of N-hydroxymethylene ibogaine. Its structure was established principally by two-dimensional NMR spectroscopy and its solid state structure was determined through X-ray diffraction analysis (Fig. 2, Fig. 4).

The conformation of this compound is stabilized by an intermolecular hydrogen bond between the hydroxyl O_2_—H_2_ group and atom N_4_ (Fig. 3).

### Experimental

The title compound (2) was prepared by reaction of ibogaine (1) (100 mg, 0.3 mmol) with formaldehyde-acetic acid solution (pH= 4) (10 ml). The mixture was stirred at room temperature for 2 h. Then, the mixture was diluted with H_2_O, made alkaline with an NH_4_OH solution (pH = 9) and immediately extracted with CH_2_Cl_2_. The organic phase was dried over sodium sulfate, filtered and concentrated under reduced pressure. The concentrate was then purified by chromatography on silica gel column with dichloromethane as eluent to yield 47% of the title compound.

N-hydroxymethylene ibogaïne (2), white crystals (CH_2_Cl_2_), C_21_H_28_N_2_O_2_: 340, m.p. 436 K, UV: λ_max_(EtOH) nm = 209, 287, 230. IR: (KBr) ν_max_(cm^-1^): 3448, 3101,2935,1617, 1586, 1482, 1456. Spectroscopic analysis, ^1^H NMR (300 MHz; CDCl_3_-d_6_, p.p.m.): 0.91 (t, J = 7.2 Hz, 3H, Me_18_); 1.26 (m, 2H, H_15_); 1.61 (m, 1H, H_19_); 1.62 (m, 1H, H_17_); 1.75 (m, 1H, H_19_); 1.83 (m, 1H, H_20_); 1.95 (m, 1H, H_14_); 2.13 (m, 1H, H_17_); 2.56 (m, 1H, H_6_); 2.89 (m, 1H, H_21_); 2.90 (m, 1H, H_16_); 2.95 (m, 1H, H_3_); 3.12 (m, 1H, H_5_); 3.26 (m, 1H, H_3_); 3.30 (m, 1H, H_6_); 3.31 (m, 1H, H_5_); 3.85 (s, 3H, CH_3_—O); 5.50 (dd, J= 11.7, 2H, N_1_—CH_2_OH); 6.83 (dd, J = 8.7, 2.4, 1H, aromatic H, H_11_); 6.90 (d, J = 2.4, 1H, aromatic H, H_9_); 7.25 (d, J = 8.7, 1H, aromatic H, H_12_). ^13^C NMR (75 MHz; CDCl_3_-d_6_, p.p.m.): 11.9, C_18_; 20.2, C_6_; 25.7, C_14_; 27.6, C_19_; 29.7, C_15_; 33.4, C_17_; 41.7, C_16_; 41.7, C_20_; 50.3, C_3_; 54.6, C_5_; 56.1, O—CH_3_; 58.2, C_21_; 66.2, N_1_—CH_2_OH; 100.9, C_9_; 109.7, C_7_; 110.2, C_12_; 111.2, C_11_; 128.9, C_8_; 142.3, C_2_; 154.5, C_10_. Repeated recrystallizations from dichloromethane afforded white crystals suitable for single crystal X-ray diffraction.

### Refinement

All H atoms were fixed geometrically and treated as riding with C—H = 0.98 Å (Cmethine), 0.97 Å (Cmethylene), 0.96 Å (Cmethyl), 0.93 Å (CH_2_) and O—H = 0.82 Å with *U*_iso_(H) = 1.2*U*_eq_(Cmethylene, methine, CH_2_) or *U*_iso_(H) = 1.5*U*_eq_(Cmethyl, O).

In the absence of anomalous scattering Friedel pairs were merged and any references to the Flack parameter were removed.

### Crystal data

**Table:**

C_21_H_28_N_2_O_2_	*D*_x_ = 1.255 Mg m^−^^3^
*M**_r_* = 340.45	Melting point: 436 K
Orthorhombic, *P*2_1_2_1_2_1_	Mo *K*α radiation λ = 0.71070 Å
Hall symbol: P 2ac 2ab	Cell parameters from 2130 reflections
*a* = 8.4990 (10) Å	θ = 3.2–24.5º
*b* = 10.2537 (11) Å	µ = 0.08 mm^−^^1^
*c* = 20.676 (3) Å	*T* = 293 (2) K
*V* = 1801.8 (4) Å^3^	Prism, colourless
*Z* = 4	0.47 × 0.33 × 0.26 mm
*F*_000_ = 736	

### Data collection

**Table:**

Bruker Kappa APEXII CCD diffractometer	2131 independent reflections
Radiation source: fine-focus sealed tube	1225 reflections with *I* > 2σ(*I*)
Monochromator: graphite	*R*_int_ = 0.079
*T* = 296 K	θ_max_ = 26.4º
φ and ω scans	θ_min_ = 2.0º
Absorption correction: multi-scan(Coppens *et al.*, 1965)	*h* = −10→10
*T*_min_ = 0.962, *T*_max_ = 0.981	*k* = −12→11
9906 measured reflections	*l* = −25→25

### Refinement

**Table:**

Refinement on *F*^2^	Hydrogen site location: inferred from neighbouring sites
Least-squares matrix: full	H-atom parameters constrained
*R*[*F*^2^ > 2σ(*F*^2^)] = 0.049	*w* = 1/[σ^2^(*F*_o_^2^) + (0.06*P*)^2^] where *P* = (*F*_o_^2^ + 2*F*_c_^2^)/3
*wR*(*F*^2^) = 0.123	(Δ/σ)_max_ < 0.001
*S* = 1.00	Δρ_max_ = 0.25 e Å^−^^3^
2131 reflections	Δρ_min_ = −0.17 e Å^−^^3^
228 parameters	Extinction correction: SHELXL97 (Sheldrick, 2008), Fc^*^=kFc[1+0.001xFc^2^λ^3^/sin(2θ)]^-1/4^
Primary atom site location: structure-invariant direct methods	Extinction coefficient: 0.013 (2)
Secondary atom site location: difference Fourier map	

### Special details

**Table:**

Geometry. Bond distances, angles etc. have been calculated using the rounded fractional coordinates. All esds are estimated from the variances of the (full) variance-covariance matrix. The cell esds are taken into account in the estimation of distances, angles and torsion angles
Refinement. Refinement of *F*^2^ against ALL reflections. The weighted *R*-factor *wR* and goodness of fit *S* are based on *F*^2^, conventional *R*-factors *R* are based on *F*, with *F* set to zero for negative *F*^2^. The threshold expression of *F*^2^ > σ(*F*^2^) is used only for calculating *R*-factors(gt) *etc*. and is not relevant to the choice of reflections for refinement. *R*-factors based on *F*^2^ are statistically about twice as large as those based on *F*, and *R*- factors based on ALL data will be even larger.

### Fractional atomic coordinates and isotropic or equivalent isotropic displacement parameters (Å^2^)

**Table:**

	*x*	*y*	*z*	*U*_iso_*/*U*_eq_	
O1	0.3015 (3)	0.7399 (3)	0.67173 (13)	0.0699 (10)	
O2	0.4685 (2)	0.3819 (3)	0.37616 (16)	0.0749 (10)	
N1	0.2906 (3)	0.5196 (3)	0.42942 (14)	0.0473 (10)	
N4	−0.2039 (3)	0.3716 (3)	0.39872 (13)	0.0444 (10)	
C2	0.1436 (4)	0.4598 (4)	0.42969 (16)	0.0438 (10)	
C3	−0.1969 (4)	0.2289 (4)	0.4092 (2)	0.0647 (16)	
C5	−0.2164 (3)	0.4504 (4)	0.45746 (15)	0.0466 (12)	
C6	−0.0883 (3)	0.4396 (4)	0.50880 (17)	0.0578 (13)	
C7	0.0731 (4)	0.4847 (4)	0.48818 (15)	0.0465 (12)	
C8	0.1799 (4)	0.5612 (4)	0.52547 (15)	0.0451 (11)	
C9	0.1706 (4)	0.6143 (4)	0.58812 (16)	0.0478 (10)	
C10	0.2951 (4)	0.6835 (4)	0.61088 (18)	0.0529 (13)	
C11	0.4272 (4)	0.7052 (4)	0.5718 (2)	0.0566 (15)	
C12	0.4392 (4)	0.6560 (4)	0.51093 (19)	0.0558 (15)	
C13	0.3148 (3)	0.5818 (3)	0.48816 (16)	0.0449 (13)	
C14	−0.0654 (4)	0.1689 (4)	0.36909 (19)	0.0612 (15)	
C15	−0.0987 (5)	0.1998 (5)	0.2990 (2)	0.0750 (18)	
C16	0.0864 (3)	0.3789 (4)	0.37486 (16)	0.0454 (12)	
C17	0.0882 (4)	0.2308 (4)	0.38900 (18)	0.0586 (13)	
C18	−0.2517 (6)	0.3734 (8)	0.1820 (3)	0.140 (4)	
C19	−0.2565 (4)	0.3954 (7)	0.2521 (2)	0.100 (3)	
C20	−0.1088 (4)	0.3479 (5)	0.28785 (18)	0.0602 (16)	
C21	−0.0813 (3)	0.4137 (4)	0.35298 (14)	0.0431 (12)	
C22	0.4069 (4)	0.5073 (4)	0.37864 (17)	0.0572 (13)	
C23	0.1784 (5)	0.7078 (5)	0.71452 (18)	0.0777 (16)	
H2	0.56220	0.38540	0.36695	0.1121*	
H3A	−0.29665	0.18987	0.39714	0.0776*	
H3B	−0.17874	0.21105	0.45467	0.0776*	
H5A	−0.31577	0.42901	0.47796	0.0558*	
H5B	−0.22240	0.54107	0.44437	0.0558*	
H6A	−0.12032	0.49033	0.54616	0.0692*	
H6B	−0.08102	0.34926	0.52234	0.0692*	
H9	0.08122	0.60219	0.61337	0.0573*	
H11	0.50966	0.75501	0.58804	0.0679*	
H12	0.52756	0.67152	0.48556	0.0670*	
H14	−0.06143	0.07430	0.37574	0.0738*	
H15A	−0.19714	0.15943	0.28629	0.0903*	
H15B	−0.01575	0.16369	0.27219	0.0903*	
H16	0.15678	0.39445	0.33813	0.0547*	
H17A	0.17411	0.19026	0.36553	0.0701*	
H17B	0.10551	0.21657	0.43483	0.0701*	
H18A	−0.34656	0.40616	0.16267	0.2104*	
H18B	−0.24268	0.28170	0.17345	0.2104*	
H18C	−0.16270	0.41811	0.16388	0.2104*	
H19A	−0.34761	0.35095	0.26980	0.1201*	
H19B	−0.26962	0.48797	0.26016	0.1201*	
H20	−0.01894	0.37112	0.26050	0.0723*	
H21	−0.08848	0.50836	0.34730	0.0517*	
H22A	0.35897	0.52830	0.33737	0.0686*	
H22B	0.49145	0.56900	0.38624	0.0686*	
H23A	0.19868	0.74605	0.75608	0.1165*	
H23B	0.17177	0.61479	0.71882	0.1165*	
H23C	0.08078	0.74087	0.69783	0.1165*	

### Atomic displacement parameters (Å^2^)

**Table:**

	*U*^11^	*U*^22^	*U*^33^	*U*^12^	*U*^13^	*U*^23^
O1	0.0692 (14)	0.082 (2)	0.0586 (15)	−0.0123 (17)	−0.0034 (16)	−0.0077 (18)
O2	0.0368 (11)	0.078 (2)	0.110 (2)	−0.0021 (13)	0.0146 (16)	−0.023 (2)
N1	0.0333 (14)	0.051 (2)	0.0577 (17)	−0.0022 (14)	0.0062 (15)	−0.0010 (17)
N4	0.0345 (12)	0.046 (2)	0.0527 (17)	−0.0025 (13)	0.0077 (14)	0.0075 (17)
C2	0.0309 (14)	0.048 (2)	0.0525 (18)	−0.0080 (15)	0.0009 (16)	0.008 (2)
C3	0.061 (2)	0.055 (3)	0.078 (3)	−0.003 (2)	0.012 (2)	0.011 (2)
C5	0.0295 (14)	0.062 (3)	0.0484 (17)	−0.0091 (15)	0.0095 (16)	0.000 (2)
C6	0.0319 (15)	0.087 (3)	0.0545 (19)	−0.0096 (18)	0.0043 (18)	0.008 (2)
C7	0.0367 (15)	0.058 (3)	0.0449 (16)	0.0021 (17)	0.0018 (17)	0.007 (2)
C8	0.0375 (16)	0.047 (2)	0.0508 (19)	0.0016 (16)	−0.0019 (16)	0.007 (2)
C9	0.0363 (14)	0.052 (2)	0.0551 (19)	0.0004 (16)	−0.0013 (17)	0.004 (2)
C10	0.0477 (17)	0.053 (3)	0.058 (2)	0.0073 (19)	−0.007 (2)	0.001 (2)
C11	0.0418 (15)	0.051 (3)	0.077 (3)	−0.0054 (19)	−0.003 (2)	−0.008 (2)
C12	0.0453 (18)	0.054 (3)	0.068 (3)	−0.0033 (18)	0.005 (2)	−0.002 (2)
C13	0.0349 (16)	0.047 (3)	0.0529 (19)	−0.0033 (16)	0.0003 (18)	0.004 (2)
C14	0.0517 (19)	0.054 (3)	0.078 (3)	0.002 (2)	0.008 (2)	0.001 (2)
C15	0.058 (2)	0.090 (4)	0.077 (3)	−0.009 (2)	−0.008 (2)	−0.022 (3)
C16	0.0328 (14)	0.058 (3)	0.0455 (17)	0.0030 (17)	0.0000 (17)	0.005 (2)
C17	0.0548 (18)	0.055 (3)	0.066 (2)	0.0097 (19)	−0.005 (2)	−0.002 (2)
C18	0.089 (4)	0.248 (9)	0.084 (4)	−0.016 (5)	−0.013 (3)	0.027 (5)
C19	0.041 (2)	0.198 (7)	0.061 (3)	−0.001 (3)	−0.0052 (19)	−0.016 (4)
C20	0.0376 (16)	0.089 (4)	0.054 (2)	0.0018 (19)	0.0005 (18)	0.002 (3)
C21	0.0339 (15)	0.052 (3)	0.0433 (16)	−0.0035 (16)	0.0012 (16)	0.009 (2)
C22	0.0332 (16)	0.081 (3)	0.0575 (19)	−0.0033 (18)	0.0057 (18)	−0.001 (3)
C23	0.077 (2)	0.105 (4)	0.051 (2)	−0.002 (3)	0.002 (2)	−0.007 (3)

### Geometric parameters (Å, °)

**Table:**

O1—C10	1.386 (5)	C20—C21	1.524 (5)
O1—C23	1.409 (5)	C3—H3A	0.9701
O2—C22	1.389 (5)	C3—H3B	0.9701
O2—H2	0.8196	C5—H5A	0.9701
N1—C2	1.392 (4)	C5—H5B	0.9696
N1—C22	1.447 (4)	C6—H6A	0.9702
N1—C13	1.387 (4)	C6—H6B	0.9697
N4—C3	1.480 (5)	C9—H9	0.9301
N4—C21	1.472 (4)	C11—H11	0.9299
N4—C5	1.463 (4)	C12—H12	0.9297
C2—C7	1.374 (5)	C14—H14	0.9803
C2—C16	1.486 (5)	C15—H15A	0.9697
C3—C14	1.522 (5)	C15—H15B	0.9702
C5—C6	1.525 (4)	C16—H16	0.9798
C6—C7	1.509 (4)	C17—H17A	0.9703
C7—C8	1.426 (5)	C17—H17B	0.9700
C8—C13	1.398 (4)	C18—H18A	0.9605
C8—C9	1.407 (5)	C18—H18B	0.9598
C9—C10	1.358 (5)	C18—H18C	0.9606
C10—C11	1.401 (5)	C19—H19A	0.9702
C11—C12	1.360 (6)	C19—H19B	0.9701
C12—C13	1.385 (5)	C20—H20	0.9797
C14—C15	1.510 (6)	C21—H21	0.9796
C14—C17	1.509 (5)	C22—H22A	0.9698
C15—C20	1.538 (7)	C22—H22B	0.9702
C16—C17	1.547 (6)	C23—H23A	0.9602
C16—C21	1.537 (4)	C23—H23B	0.9595
C18—C19	1.467 (8)	C23—H23C	0.9604
C19—C20	1.536 (6)		
			
O2···C3^i^	3.319 (4)	H5A···O2^iv^	2.8329
O2···C5^i^	3.239 (4)	H5A···H2^iv^	2.5581
O2···N4^i^	2.825 (3)	H5A···H3B	2.5657
O2···C16	3.248 (3)	H5A···H17B^v^	2.4346
O1···H20^ii^	2.8425	H5B···H21	2.3315
O1···H21^iii^	2.7735	H5B···C10^vii^	3.0501
O2···H3A^i^	2.8371	H5B···C11^vii^	2.9150
O2···H5A^i^	2.8329	H6A···C9	2.9124
O2···H16	2.7665	H6A···H9	2.4861
O2···H19A^i^	2.7165	H6B···C3	2.8223
N4···O2^iv^	2.825 (3)	H6B···H3B	2.1577
N4···H2^iv^	2.0984	H9···C6	3.0870
N4···H19A	2.9397	H9···C23	2.4957
C3···O2^iv^	3.319 (4)	H9···H6A	2.4861
C3···C8^v^	3.431 (6)	H9···H23B	2.3157
C5···O2^iv^	3.239 (4)	H9···H23C	2.2520
C8···C3^vi^	3.431 (6)	H9···H18A^ix^	2.2414
C16···O2	3.248 (3)	H12···C22	2.9621
C3···H2^iv^	2.7441	H12···H22B	2.3273
C3···H6B	2.8223	H12···C8^iii^	3.0396
C5···H2^iv^	2.7362	H12···C9^iii^	2.9363
C6···H9	3.0870	H14···C9^v^	3.0798
C6···H3B	2.7084	H14···C10^v^	2.9242
C7···H17B	2.9751	H14···C11^v^	3.0658
C8···H3A^vi^	3.0377	H15A···H3A	2.4629
C8···H12^vii^	3.0396	H15A···H19A	2.3682
C8···H3B^vi^	3.0667	H15B···H17A	2.5303
C9···H23B	2.7024	H16···O2	2.7665
C9···H23C	2.7226	H16···C15	3.0582
C9···H6A	2.9124	H16···C22	2.5611
C9···H14^vi^	3.0798	H16···H20	2.2054
C9···H12^vii^	2.9363	H16···H22A	2.1993
C10···H5B^iii^	3.0501	H16···C23^x^	3.0973
C10···H14^vi^	2.9242	H16···H23A^x^	2.5422
C11···H5B^iii^	2.9150	H17A···H15B	2.5303
C11···H14^vi^	3.0658	H17A···H23A^x^	2.5916
C11···H18C^ii^	3.0380	H17B···C7	2.9751
C12···H22B	2.7640	H17B···H3B	2.4511
C15···H16	3.0582	H17B···H5A^vi^	2.4346
C15···H18B	2.9902	H18A···H9^viii^	2.2414
C16···H22A	2.8834	H18B···C15	2.9902
C18···H23C^viii^	3.0500	H18C···H20	2.3908
C19···H2^iv^	2.8326	H18C···C11^x^	3.0380
C21···H2^iv^	3.0574	H19A···O2^iv^	2.7165
C22···H12	2.9621	H19A···N4	2.9397
C22···H16	2.5611	H19A···H2^iv^	2.1788
C23···H9	2.4957	H19A···H15A	2.3682
C23···H16^ii^	3.0973	H19A···C23^viii^	3.0941
C23···H19A^ix^	3.0941	H19B···H21	2.3791
H2···N4^i^	2.0984	H20···H16	2.2054
H2···C3^i^	2.7441	H20···H18C	2.3908
H2···C5^i^	2.7362	H20···O1^x^	2.8425
H2···C19^i^	2.8326	H21···H5B	2.3315
H2···C21^i^	3.0574	H21···H19B	2.3791
H2···H3A^i^	2.4183	H21···O1^vii^	2.7735
H2···H5A^i^	2.5581	H22A···C16	2.8834
H2···H19A^i^	2.1788	H22A···H16	2.1993
H3A···O2^iv^	2.8371	H22B···C12	2.7640
H3A···H2^iv^	2.4183	H22B···H12	2.3273
H3A···H15A	2.4629	H23A···H16^ii^	2.5421
H3A···C8^v^	3.0377	H23A···H17A^ii^	2.5916
H3B···C6	2.7084	H23B···C9	2.7024
H3B···H5A	2.5657	H23B···H9	2.3157
H3B···H6B	2.1577	H23C···C9	2.7226
H3B···H17B	2.4511	H23C···H9	2.2520
H3B···C8^v^	3.0667	H23C···C18^ix^	3.0500
			
C10—O1—C23	116.3 (3)	C5—C6—H6A	108.37
C22—O2—H2	109.52	C5—C6—H6B	108.42
C2—N1—C13	109.4 (3)	C7—C6—H6A	108.40
C2—N1—C22	125.3 (3)	C7—C6—H6B	108.44
C13—N1—C22	125.0 (3)	H6A—C6—H6B	107.47
C3—N4—C5	115.3 (3)	C8—C9—H9	120.72
C3—N4—C21	110.8 (3)	C10—C9—H9	120.77
C5—N4—C21	115.0 (3)	C10—C11—H11	118.88
N1—C2—C16	122.4 (3)	C12—C11—H11	118.78
C7—C2—C16	129.2 (3)	C11—C12—H12	121.28
N1—C2—C7	108.3 (3)	C13—C12—H12	121.25
N4—C3—C14	110.4 (3)	C3—C14—H14	110.42
N4—C5—C6	119.1 (3)	C15—C14—H14	110.40
C5—C6—C7	115.5 (3)	C17—C14—H14	110.38
C2—C7—C8	107.5 (3)	C14—C15—H15A	109.40
C6—C7—C8	126.5 (3)	C14—C15—H15B	109.39
C2—C7—C6	126.0 (3)	C20—C15—H15A	109.43
C7—C8—C13	107.9 (3)	C20—C15—H15B	109.37
C9—C8—C13	119.7 (3)	H15A—C15—H15B	108.01
C7—C8—C9	132.4 (3)	C2—C16—H16	107.50
C8—C9—C10	118.5 (3)	C17—C16—H16	107.47
O1—C10—C9	124.3 (3)	C21—C16—H16	107.47
O1—C10—C11	115.2 (3)	C14—C17—H17A	109.54
C9—C10—C11	120.5 (3)	C14—C17—H17B	109.54
C10—C11—C12	122.3 (3)	C16—C17—H17A	109.49
C11—C12—C13	117.5 (3)	C16—C17—H17B	109.50
N1—C13—C8	107.0 (3)	H17A—C17—H17B	108.06
C8—C13—C12	121.4 (3)	C19—C18—H18A	109.51
N1—C13—C12	131.6 (3)	C19—C18—H18B	109.56
C3—C14—C15	107.5 (3)	C19—C18—H18C	109.50
C15—C14—C17	109.6 (3)	H18A—C18—H18B	109.45
C3—C14—C17	108.5 (3)	H18A—C18—H18C	109.38
C14—C15—C20	111.2 (4)	H18B—C18—H18C	109.44
C2—C16—C17	113.6 (3)	C18—C19—H19A	108.82
C2—C16—C21	113.5 (3)	C18—C19—H19B	108.86
C17—C16—C21	107.0 (3)	C20—C19—H19A	108.77
C14—C17—C16	110.7 (3)	C20—C19—H19B	108.75
C18—C19—C20	113.8 (4)	H19A—C19—H19B	107.64
C15—C20—C21	107.2 (3)	C15—C20—H20	106.43
C19—C20—C21	114.2 (4)	C19—C20—H20	106.40
C15—C20—C19	115.5 (4)	C21—C20—H20	106.43
N4—C21—C16	113.5 (3)	N4—C21—H21	108.88
C16—C21—C20	107.4 (3)	C16—C21—H21	108.85
N4—C21—C20	109.2 (3)	C20—C21—H21	108.85
O2—C22—N1	111.4 (3)	O2—C22—H22A	109.35
N4—C3—H3A	109.57	O2—C22—H22B	109.29
N4—C3—H3B	109.56	N1—C22—H22A	109.39
C14—C3—H3A	109.57	N1—C22—H22B	109.36
C14—C3—H3B	109.57	H22A—C22—H22B	107.99
H3A—C3—H3B	108.08	O1—C23—H23A	109.46
N4—C5—H5A	107.53	O1—C23—H23B	109.51
N4—C5—H5B	107.56	O1—C23—H23C	109.47
C6—C5—H5A	107.52	H23A—C23—H23B	109.49
C6—C5—H5B	107.55	H23A—C23—H23C	109.41
H5A—C5—H5B	107.04	H23B—C23—H23C	109.48
			
C23—O1—C10—C9	8.4 (6)	C6—C7—C8—C13	−179.5 (3)
C23—O1—C10—C11	−173.2 (4)	C6—C7—C8—C9	0.3 (7)
C13—N1—C2—C16	−176.6 (3)	C2—C7—C8—C13	0.2 (4)
C13—N1—C2—C7	0.6 (4)	C7—C8—C13—C12	178.5 (3)
C22—N1—C2—C7	174.5 (3)	C7—C8—C13—N1	0.2 (4)
C2—N1—C13—C12	−178.5 (4)	C13—C8—C9—C10	−0.7 (6)
C22—N1—C13—C12	7.5 (6)	C7—C8—C9—C10	179.5 (4)
C2—N1—C22—O2	−68.4 (4)	C9—C8—C13—N1	−179.7 (3)
C13—N1—C22—O2	104.6 (4)	C9—C8—C13—C12	−1.4 (5)
C22—N1—C2—C16	−2.7 (6)	C8—C9—C10—C11	2.3 (6)
C22—N1—C13—C8	−174.4 (3)	C8—C9—C10—O1	−179.5 (4)
C2—N1—C13—C8	−0.5 (4)	O1—C10—C11—C12	179.7 (4)
C5—N4—C21—C16	74.8 (4)	C9—C10—C11—C12	−1.8 (6)
C3—N4—C21—C16	−58.2 (4)	C10—C11—C12—C13	−0.2 (6)
C5—N4—C21—C20	−165.3 (3)	C11—C12—C13—C8	1.8 (5)
C5—N4—C3—C14	−133.2 (3)	C11—C12—C13—N1	179.6 (4)
C21—N4—C3—C14	−0.3 (4)	C17—C14—C15—C20	−59.4 (4)
C21—N4—C5—C6	−72.2 (4)	C3—C14—C17—C16	−62.3 (4)
C3—N4—C21—C20	61.6 (4)	C3—C14—C15—C20	58.3 (4)
C3—N4—C5—C6	58.7 (4)	C15—C14—C17—C16	54.8 (4)
N1—C2—C7—C6	179.2 (3)	C14—C15—C20—C19	−128.7 (3)
C7—C2—C16—C17	−70.8 (5)	C14—C15—C20—C21	−0.1 (4)
N1—C2—C16—C17	105.7 (4)	C2—C16—C21—C20	166.7 (3)
C16—C2—C7—C8	176.5 (4)	C17—C16—C21—N4	53.7 (4)
N1—C2—C16—C21	−131.7 (3)	C2—C16—C21—N4	−72.4 (4)
C16—C2—C7—C6	−3.8 (7)	C2—C16—C17—C14	133.0 (3)
N1—C2—C7—C8	−0.5 (4)	C17—C16—C21—C20	−67.2 (4)
C7—C2—C16—C21	51.7 (5)	C21—C16—C17—C14	6.9 (4)
N4—C3—C14—C17	59.5 (4)	C18—C19—C20—C21	157.5 (5)
N4—C3—C14—C15	−59.0 (4)	C18—C19—C20—C15	−77.5 (6)
N4—C5—C6—C7	64.4 (5)	C19—C20—C21—N4	68.8 (5)
C5—C6—C7—C8	137.5 (4)	C19—C20—C21—C16	−167.7 (4)
C5—C6—C7—C2	−42.2 (6)	C15—C20—C21—C16	63.0 (4)
C2—C7—C8—C9	−180.0 (4)	C15—C20—C21—N4	−60.5 (4)

Symmetry codes: (i) *x*+1, *y*, *z*; (ii) −*x*+1/2, −*y*+1, *z*+1/2; (iii) *x*+1/2, −*y*+3/2, −*z*+1; (iv) *x*−1, *y*, *z*; (v) *x*−1/2, −*y*+1/2, −*z*+1; (vi) *x*+1/2, −*y*+1/2, −*z*+1; (vii) *x*−1/2, −*y*+3/2, −*z*+1; (viii) −*x*−1/2, −*y*+1, *z*−1/2; (ix) −*x*−1/2, −*y*+1, *z*+1/2; (x) −*x*+1/2, −*y*+1, *z*−1/2.

### Hydrogen-bond geometry (Å, °)

**Table:**

*D*—H···*A*	*D*—H	H···*A*	*D*···*A*	*D*—H···*A*
O2—H2···N4^i^	0.8200	2.1000	2.825 (3)	148.00

Symmetry codes: (i) *x*+1, *y*, *z*.
